# The Treatment of Cough in the Phthisical

**Published:** 1899-02

**Authors:** 


### THE TREATMENT OF COUGH IN THE PHTHISICAL.

AMONG the most prominent symptoms in phthisical cases is the irritating, harassing cough which is not only extremely annoying, but, if unrelieved, contributes greatly to the loss of flesh and strength by robbing the patient of rest at night. While the remedies that have been recommended from time to time are almost innumerable, physicians have usually been compelled in the end to rely chiefly upon opium or some of its derivatives. Most of these drugs, however, have the injurious property of weakening the respiratory apparatus, of diminishing the expectorating power, and of giving rise to disagreeable after-effects These objectionable features are said to be entirely absent in heroin, a new remedy which combines the sedative action of morphine and codeine with apparent freedom from injurious or unpleasant after-effects. Heroin exerts a direct influence upon the respiratory tract, increasing the volume of inspiration as well as the force of expiration, and reducing the frequency of respirations. It thus acts as a true respiratory sedative, and relieves cough without the least risk of blocking the secretions in the air passages. In tuberculous cases it has the additional effect of reducing the temperature and relieving night sweats.—*Clinical Notes.*

